# The Long‐Term Impact of Adolescent Community Weapon‐Related Violence Exposure on Depression: Insomnia as a Mediating Pathway

**DOI:** 10.1002/jcop.70121

**Published:** 2026-06-22

**Authors:** Esther Lee, Justin Heinze

**Affiliations:** ^1^ Department of Health Behavior and Health Equity University of Michigan School of Public Health, University of Michigan Ann Arbor Michigan USA

**Keywords:** adolescent, community violence, depression, insomnia, mental health, risk factors, violence

## Abstract

Adolescents exposed to community weapon‐related violence face increased mental health risks, including depression, with insomnia linked to negative mental health outcomes. Given the high rates of insomnia in this population, this study explores insomnia as a mediator between community weapon‐related violence and adult depression. Using data from the National Longitudinal Study of Adolescent to Adult Health, we analyzed the association between adolescent weapon‐related violence (Wave 1) and depression in adulthood (Wave 4), with insomnia (Wave 2) as a mediator (*N* = 3924). Community weapon‐related violence was associated with higher levels of depression in adulthood, 13 years post‐exposure, through insomnia (*β* = 0.01, *p* = 0.01). Insomnia accounted for 7% of the total effect of weapon‐related violence on depressive symptoms, indicating a partial mediation effect. Our findings identify a pathway through which adolescent community weapon‐related violence leads to depression in adulthood, underscoring the need for early interventions targeting insomnia to reduce long‐term depression risk in this population.

## 1 Weapon‐Related Violence Exposure and Adolescence

Exposure to violence (ETV) is a common experience for the 13 million adolescents (ages 15–17) in the United States (Kids Count Data Center 2023). Nearly 68% of adolescents aged 17 and younger are exposed to at least one form of violence (Finkelhor et al. 2015). The four categories of community ETV include (1) victimization (being the subject of intentional acts initiated by another person to cause harm), (2) witnessing (seeing an event involving a threat or an actual injury or death), (3) perpetration, and (4) hearing about violence (learning about someone else's victimization) (Buka et al. 2001). In this study, we focus on community‐based interpersonal violence, specifically weapon‐related (e.g., knife, gun) violence, which occurs within communities, neighborhoods, and public spaces where individuals interact daily. Moreover, this study aims to examine how community‐level exposure to weapon‐related violence—namely, through victimization or witnessing in adolescence—influences insomnia and later development of depression in adulthood.

Adolescence is a sensitive developmental phase characterized by significant neurological, physical, social, and emotional changes that impact both neural pathways and behavior patterns (Best and Ban 2021; Silvers 2022). These developmental transitions can heighten adolescents' sensitivity to new stressors, such as violent victimization, which increases from adolescence into early adulthood (Lambert et al. 2005; Morgan and Oudekerk 2019). Violent exposure, particularly when repeated, can be highly stressful and traumatic. While violent victimization in general is linked to adverse mental health outcomes, including PTSD, depression, anxiety, and substance use disorders, emerging research comparing weapon‐related violence to non‐weapon‐related violence has found that weapon‐related violence, especially involving firearms, is associated with higher psychological distress, PTSD symptoms, depression, and anxiety. Perceptions of weapon lethality and the fear that oneself or loved ones might be caught in a crossfire can contribute to the onset or exacerbation of emotional trauma (Kagawa et al. 2020; Opara et al. 2020). Drawing on the Transactional Model of Stress and Coping (Lazarus and Folkman 1984), such exposure can overwhelm adolescents' ability to process the trauma, especially when they lack coping resources and skills, disrupting emotional regulation and increasing vulnerability to mental health problems (Allwood et al. 2023; Covey et al. 2020; Kagawa et al. 2018, 2020; Langton and Truman 2014; Lee et al. 2024; Mitchell et al. 2015). For example, Mitchell et al. (2015) found that victimization involving a high lethality risk (e.g., gun or knife) was linked to more severe mental health symptoms. Kagawa et al. (2020) reported that nearly 40% of firearm victims experienced severe distress, compared to just 25% of those exposed to non‐firearm violence. These exposures can disrupt the typical developmental trajectory by influencing cognitive and behavioral growth, hindering a smooth transition to adulthood, and ultimately affecting long‐term mental and physical health and quality of life (Jain and Cohen 2013; Lee et al. 2025; Lefebvre et al. 2021; Silvers 2022). Given the nascent literature distinguishing weapon‐related from non‐weapon‐related violence, further investigation is warranted to understand the unique associations and mechanisms linking weapon‐related violence exposure to mental health. Elucidating the pathway between adolescent weapon‐related violence and adult depression can inform interventions that aim to reduce the long‐term psychological sequelae in adolescents exposed to community weapon‐related violence, recognizing that resilience factors are also critical components to address. Thus, we draw on the broader literature on violence exposure to inform our understanding of weapon‐related impacts on depression, while acknowledging the shared pathways to mental health outcomes across different types of community violence.

## 2 Adolescent Weapon‐Related Violence and Depression

Studies have consistently shown that adolescent exposure to violence has been associated with depression in adolescence and later life (Abba‐Aji et al. 2024; Heinze et al. 2018; Leibbrand et al. 2020; Semenza et al. 2021, 2025). In particular, adolescent community violence exposure has been associated with internalizing behaviors such as depression and anxiety (Fowler et al. 2009; Leibbrand et al. 2020; Mrug and Windle 2010). Fatal shootings increase the usage of antidepressants among local adolescents by 21% (Rossin‐Slater et al. 2020). Among adolescents who saw or heard gun violence, 58% reported being very or extremely afraid, sad, or upset because of the indirect gun violence exposure (Mitchell et al. 2021). Exposure to weapon‐related violence in adolescence may exacerbate the onset, severity, and duration of depression. Thus, it is important to investigate the effects of weapon‐related violence exposure on depression in early adulthood since depression often manifests in that developmental period (Schubert et al. 2017).

## 3 Adolescent Violence and Insomnia Symptoms

Adolescent exposure to community violence, whether direct or indirect is positively associated with sleep problems, including short sleep duration, poor sleep quality, and sleep–wake difficulties (de Zambotti et al. 2018; Kliewer and Lepore 2015; Rubens et al. 2014; Semenza et al. 2024; Spilsbury et al. 2014; Wright et al. 2017). In particular, insomnia, defined by the Diagnostic and Statistical Manual of Mental Disorders, Fifth Edition (DSM‐5) as a persistent difficulty with sleep initiation, maintenance, and early morning wakefulness, is notably common among adolescents who have experienced violence (American Psychiatric Association 2013). Exposure to community violence can be a traumatic event, generating a hyperarousal state, thus contributing to difficulties in sleep onset and maintenance among adolescents (Kliewer and Lepore 2015; Wright et al. 2017). Kliewer and Lepore (2015) found that among urban middle school students, witnessing violence contributed to higher insomnia (sleep–wake difficulties) after 1 year of experiencing violence.

While numerous studies investigate the broader influence of adolescent community violence exposure on sleep‐related outcomes, there is limited literature on the specific impact of weapon‐related violence. Additionally, most studies examining the relationship between adolescent community violence and sleep problems do not specifically focus on insomnia. Instead, they often address broader sleep disturbances without distinguishing the unique impact of insomnia. Moreover, many of these studies are cross‐sectional, limiting the ability to understand how insomnia develops and changes over time following exposure to weapon‐related violence.

## 4 Insomnia Symptoms as a Mediator Between Exposure to Violence and Depression

Although understudied, previous research have documented links between community violence exposure, quality of sleep, and adolescent mental health (Aiyer et al. 2014; Dziurkowska and Wesolowski 2021; Semenza et al. 2021, 2023, 2024, 2025). Early exposure to weapon‐related violence could influence mental health by first disrupting adolescent sleep patterns, which in turn manifest as mental health symptoms. Insomnia is a common response to traumatic events (e.g., exposure to violence) and can disrupt the normal processing of traumatic events, exacerbating stress and anxiety (Kajeepeta et al. 2015). Chronic insomnia can lead to neurobiological changes that increase vulnerability to depression (Baglioni et al. 2010). Adolescents who experience community violence are at a higher risk for developing persistent sleep problems, which can potentially contribute to the onset and maintenance of depressive symptoms over time (Wright et al. 2017). For example, Heissel et al. (2018) found that exposure to local violent crime led to later bedtimes and disrupted sleep, with heightened cortisol levels upon waking, particularly when crimes occurred in close proximity to adolescents' homes (Heissel et al. 2018). One plausible pathway documented by studies is that stress from exposure to violence can elevate adolescent stress through overactivation of the hypothalamic‐pituitary‐adrenal (HPA) axis, leading to increased release of stress hormones like cortisol and disruption of sleep patterns (Aiyer et al. 2014; Finlay et al. 2022; Ford and Browning 2014; Geronimus 2021; Geronimus et al. 2006; Wright et al. 2017; Yao et al. 2019). Moreover, consistent or persistent exposure likely exacerbates the amount of sleep disruption, increasing the likelihood of depression (O'Leary et al. 2017; Spilsbury et al. 2014). Insomnia has also been shown to impair emotional regulation and cognitive functioning, both of which are critical in coping with traumatic experiences and mitigating the long‐term effects of such exposure, such as depression (Blake et al. 2018; Vanek et al. 2020). By examining insomnia as a mediator, we can gain a more nuanced understanding of the underlying pathways linking adolescent exposure to community violence and adult depression, thereby informing targeted interventions that address insomnia to mitigate depression in adulthood.

The present research aimed to examine the relationship between exposure to community weapon‐related violence in adolescence, insomnia, and depressive symptoms in adulthood. It is predicted that insomnia would mediate the relationship between exposure to violence in adolescence at Wave 1 and later depression in adulthood in Wave 4. To test our hypothesis, we used three waves of the National Longitudinal Study of Adolescent to Adult Health (Add Health) to examine the direct effect of adolescent exposure to violence on depressive symptoms reported 13 years later, as well as the indirect effect of this exposure on adulthood depression through insomnia.

## 5 Methods

The current study employs data from three waves of the National Longitudinal Study of Adolescent to Adult Health (Add Health), a nationally representative sample of adolescents in grades 7–12 who were followed from 1994 to 1995 into young adulthood (ages 24–32) in 2008. Wave 1 was collected in 1994–1995 through in‐school questionnaires and in‐home interviews. A 45‐min in‐school questionnaire was administered to 90,118 students in grades 7–12 from 145 different middle, junior, and high schools. Among the students who completed the in‐school questionnaires, 20,746 adolescents and their caregivers were sampled to participate in an in‐home interview that included topics such as health status, sexual activity, criminal activities, and substance use. The in‐home questionnaire was administered through the Audio Computer‐Assisted Self Interview (ACASI) to address the sensitive nature of these questions, including questions about the exposure variable of interest, weapon‐related violence, which included firearm‐ and knife‐related violence, and depression. The mediator variable, insomnia, was taken from Wave 2 in 1996. The outcome variable, adult depression, was measured in Wave 4, collected from the sample in 2008 when the respondents were ages 24–32.

Missing data patterns were assessed in two ways: by respondent attrition across waves and by completion of at least one baseline violence item. At the respondent‐ID level, 25.7% of the baseline sample was lost by Wave 2 and 18.8% of Wave 2 participants were lost by Wave 4. Among those with at least one baseline violence response, 59.9% had violence data in all three waves. We used chi‐squared tests and logistic regression to examine associations between baseline characteristics and dropout. Chi‐squared tests identified significant differences by sex only (males more likely to drop out than females), while multivariable logistic regression showed no significant predictors of missingness. Based on these patterns, we assumed data were missing at random (MAR) and used multiple imputation using the Predictive Mean Matching (PMM) method within the *MICE* package in R (Arbuckle 1996; Enders and Bandalos 2001; Knol et al. 2010). Multiple imputation was applied to participants with data on at least one of Waves 1, 2, or 4 and valid survey weights in accordance with Add Health analytic guidelines, allowing individuals with partial data to be included in the analysis to preserve sample size and improve the representativeness. Ten imputed datasets were generated, and the estimates were pooled to account for the variability between imputations, yielding a final analytic sample of 3924 adolescents. This study was submitted to the Institutional Review Board (IRB) and was subsequently deemed Not Regulated.

To utilize the longitudinal nature of the sample, we measured community weapon‐related violence at Wave 1 and examined its associations with insomnia at Wave 2 and depression at Wave 4. Given documented differences in depression and insomnia rates by age, sex, race/ethnicity, household income, parents' education, and substance use (i.e., cigarettes, alcohol), we included these variables as controls, along with baseline measures of depression and insomnia at Wave 1 (Hankin and Abramson 2001; Parker and Brotchie 2010; Phiri et al. 2023; Ranaei et al. 2022; Twenge and Nolen‐Hoeksema 2002; Wade et al. 2002).

## 6 Measures

### 6.1 Depressive Symptoms (Wave 4)

Depressive symptoms in adulthood were assessed at Wave 4 using seven retained items from the modified 9‐item Center for Epidemiologic Studies Depression Scale (CES‐D‐9), after excluding two items that conceptually overlapped with insomnia. The CES‐D‐9 has previously demonstrated high reliability and validity among adolescent populations (Andresen et al. 1994; Bradley et al. 2010; Desch et al. 2023). The CES‐D‐9 items assessed the frequency of participants’ feelings in the past 7 days, including “you were bothered by things that don't usually bother you,” “you felt depressed,” “you were too tired to do things,” and “you felt sad.” We also excluded two items that overlapped with insomnia. For example, we excluded “you felt that you were too tired to do things” and “you had trouble keeping your mind on what you were doing”. Items positively worded, such as “you felt you were just as good as other people” and “you enjoyed life,” were reverse‐coded. Response options were on a 4‐point Likert scale: never or rarely, sometimes, a lot of times, and most of the time or all the time. The seven retained items were summed to create a depressive symptom score ranging from 0 to 21, with higher scores indicating greater depressive symptoms. The Cronbach's alpha was 0.81. Depression items in Wave 1 were also assessed as a covariate in the analysis.

### 6.2 Community Weapon‐Related Violence Exposure (Wave 1)

Community weapon‐related violence during adolescence was measured using four items in Wave 1, assessing violence victimization (involving both firearms and knives) over the past 12 months. Participants were asked how often the following events occurred: “you saw someone shoot or stab another person,” “someone pulled a knife or gun on you,” “someone shot you,” and “someone cut or stabbed you.” Response options ranged from 0 = never, 1 = once, and 2 = more than once. The four victimization items were summed to create a total score and then dichotomized (0 = no exposure; 1 = any exposure) to account for the high prevalence of respondents reporting no victimization.

### 6.3 Insomnia Symptoms (Wave 2)

Insomnia was assessed in Wave 2 using a single‐item measure that asked respondents to report the frequency of difficulty “falling or staying asleep” in the past 12 months. Response options included: (0) never, (1) just a few times, (2) about once a week, (3) almost every day, and (4) every day. Although insomnia was measured as a single item, it captures the key aspects of insomnia disorder, that is, difficulty with sleep initiation and maintenance, as defined by the fifth edition of the Diagnostic and Statistical Manual of Mental Disorders (DSM‐5) and supported by previous Add Health studies (American Psychiatric Association 2013; Desch et al. 2023; Li et al. 2019; Rojo‐Wissar et al. 2020, 2021; Semenza et al. 2024).

### 6.4 Control Variables

Age was assessed from the participant's birth date (between 10 and 19 years). Additional covariates in Wave 1 included sex (male or female); minority status (Non‐Hispanic White vs. Minority, defined here as Black/African American and Hispanic participants only); household income in 1994 (ranging in thousands of dollars between $0 and $999); father's highest educational attainment (biological, step‐, foster‐, or adoptive); smoking (days smoked in the past 30 days, 0–30); and alcohol use frequency in the past 12 months was assessed on a 7‐point scale ranging from daily to never. Depressive symptoms and insomnia at Wave 1 were included as control variables.

### 6.5 Statistical Analysis

We assessed descriptive analyses for demographic characteristics, including means and standard deviations for continuous variables and percentages for categorical factors, as well as correlations. Missing data were handled with multiple imputation, and derived variables were created as previously described. Prior to mediation models, bivariate relationships among the primary continuous variables were examined using Pearson correlation coefficients; the correlation matrix is presented in Supporting Information S1: Table 1. The mediation analysis was conducted using one independent variable (exposure to community weapon‐related violence), one dependent variable (depressive symptoms), and the mediator variable (insomnia symptoms). The analysis controlled for Wave 1 depressive symptoms, Wave 1 insomnia, age, sex, race/ethnicity, household income, father's highest education, and substance use (assessed in Waves 1, 2, and 4). In Model 1, we examined the direct effect of exposure to weapon‐related violence on adult depressive symptoms. In Model 2, we assessed the relationship between adolescent exposure to weapon‐related violence and the mediator (insomnia in Wave 2). Finally, in Model 3, we included both exposure to weapon‐related violence and insomnia to assess the indirect effect of exposure to violence on adult depressive symptoms through insomnia. The mediation model was evaluated using the nonparametric bootstrap method to measure the indirect effect at the 95% confidence interval. Ten thousand bootstrap samples were conducted, and the significance of the mediation was assessed by examining whether the bootstrapped confidence intervals excluded zero, indicating significant mediation. All analyses were performed using the *Mediation* package in R (v4.3.1) (Tingley et al. 2014).

## 7 Results

The final analytic sample consisted of 3924 participants. Table 1 presents descriptive statistics for all variables; key characteristics are summarized below. In Wave 1, participants' mean age was *M* = 14.46 years (SD = 1.54, range = 11–19). More than half (54.7%) of the sample were females. A majority of participants (59.4%) identified as Non‐Hispanic White, while 40.6% identified as a minority, including Black/African American or Hispanic adolescents. For paternal figures, 32.6% were high school graduates; 26.0% had a bachelor's degree; 14.0% had postgraduate education; 13.8% had less than a high school education; and 13.5% had some college. The mean annual household income was $47,830 (SD = 55,420, range = 0–999,999). In Wave 1, 80.9% of participants reported no community violence exposure, and 19.1% reported one or more incidents. Depressive symptoms averaged *M* = 4.12 (SD = 3.43, range = 0–21) in Wave 1 and decreased to *M* = 3.53 (SD = 3.36, range = 0–21) by Wave 4. In Wave 2, 34.7% reported never, 41.0% rarely, 16.3% occasionally, 6.3% often, and 1.7% every day on the insomnia symptoms scale (range = 0–4). In Wave 4, alcohol use averaged *M* = 2.80 (SD = 1.59, range = 1–7), and cigarette smoking averaged *M* = 8.28 days (SD = 12.70, range = 0–30) in the past 30 days. Insomnia in Wave 2 was significantly positively correlated with depressive symptoms in Wave 4 (*r* = 0.11, *p* < 0.001); see Table 1 for the correlation matrix.

**TABLE 1:** Descriptive statistics of study sample (*N* = 3924).

Demographic variables	Wave	% (*N*)/Mean (SD)	Range
Sex			
Male	1	45.3% (1776)	
Female	1	54.7% (2148)	
Age (years)	1	14.46 (1.54)	11–19
Minority status			
Non‐Hispanic White	1	59.4% (2330)	
Minority	1	40.6% (1594)	
Household income ($)	1	47,830 (55,420)	0–999,999
Father's highest education	1		
Less than high school	1	13.8% (542)	
High school graduate	1	32.6% (1279)	
Some college	1	13.5% (531)	
Bachelor's degree	1	26.0% (1022)	
Postgraduate education	1	14.0% (550)	
Community weapon‐related violence exposure	1		
None		80.9% (3176)	
1+ incidents	1	19.1% (748)	
Depressive symptoms			
Depressive symptoms	1	4.12 (3.43)	0–21
Depressive symptoms	4	3.53 (3.36)	0–21
Insomnia symptoms			
Never	1	42.3% (1661)	0–4
Rarely	1	24.1% (944)	0–4
Occasionally	1	17.3% (679)	0–4
Often	1	10.6% (416)	0–4
Everyday	1	5.7% (224)	0–4
Never	2	34.7% (1362)	0–4
Rarely	2	41.0% (1607)	0–4
Occasionally	2	16.3% (641)	0–4
Often	2	6.3% (246)	0–4
Everyday	2	1.7% (68)	0–4
Substance use			
Alcohol use (past year)	1	5.33 (1.41)	1–7
Alcohol use (past year)	2	4.88 (1.40)	1–7
Alcohol use (past year)	4	2.80 (1.59)	1–7
Smoking (days/30 days)	1	6.73 (10.74)	0–30
Smoking (days/30 days)	2	9.00 (11.86)	0–30
Smoking (days/30 days)	4	8.28 (12.70)	0–30

*Note:* Descriptive statistics for variables from Waves 1, 2, and 4. Column percentages (with Ns) are presented for categorical variables and means (SD) for continuous variables; ranges are provided when applicable. Household income was reported in dollars for 1994. Father's education includes biological, step‐, foster‐, or adoptive. Minority status includes respondents identifying as Black/African American or Hispanic. Weapon‐related violence exposure has been coded as none versus one or more incidents in the past 12 months. Depressive symptoms are a summed score (range = 0–21). Insomnia is measured as frequency on a 0–4 scale (higher = more frequent). Alcohol use reflects past‐year drinking frequency (1 = every day or almost every day to 7 = never). Smoking is days smoked in the past 30 days (0–30).

### 7.1 Mediation Analysis

We conducted a mediation analysis to examine the relationship between exposure to community weapon‐related violence in adolescence (Wave 1), insomnia (Wave 2), and depression in adulthood (Wave 4); the standardized path model is illustrated in Supporting Information S1: Figure 1. Table 2 presents the standardized estimates as well as the direct, indirect, and total effects. In Model 1, adolescent weapon‐related violence was significantly associated with depression (*β* = 0.04, *p* = 0.03) after adjusting for covariates. In Model 2, any weapon‐related violence exposure during adolescence was associated with higher insomnia symptoms 1 year later compared to no exposure (*β* = 0.05, *p* = 0.003), adjusting for covariates. In Model 3, weapon‐related violence (*β* = 0.04, *p* = 0.03) was significantly associated with depression after adding insomnia. Further, insomnia (*β* = 0.06, *p* < 0.001) had a significant, positive association with depression, adjusting for the covariates. A summary of estimates for variables related to the outcome, depression (Wave 4), is provided in Supporting Information S1: Table 2.

**TABLE 2:** Standardized direct, indirect, and total effects of weapon‐related violence exposure on depression via insomnia.

	Model	Std. estimate	95% CI	*p*
Violence exposure (W1) → Depression (W4)	Model 1	0.04	[0.01, 0.08]	0.03*
Violence exposure (W1) → Insomnia (W2)	Model 2	0.05	[0.02, 0.08]	0.003**
Violence exposure (W1) + Insomnia (W2) → Depression (W4)	Model 3	0.04	[0.00, 0.07]	0.03*
Violence exposure (W1) + Insomnia (W2) → Depression (W4)	Model 3	0.06	[0.03, 0.09]	< 0.001***
Indirect		0.01	[0.00, 0.02]	0.01*
Direct		0.09	[0.00, 0.19]	0.03*
Total effect		0.10	[0.01, 0.20]	0.02*
Proportion mediated		0.07	[0.00, 0.39]	0.03*

*Note:* Path coefficients for *a* (weapon exposure → insomnia; Model 2), *b* (insomnia → depression controlling for exposure; Model 3), *c′* (direct effect from exposure to depression adjusting for insomnia; Model 3), and *c* (total effect of exposure on depression without the mediator; Model 1) are standardized; insomnia and depression were z‐scored prior to mediation. The indirect effect (*a × b*) was estimated via nonparametric bootstrap. Models adjust for age, minority status, household income, father's education, smoking, and alcohol. Confidence intervals are 95%; estimates are rounded (actual lower bounds > 0). Significance indicators: *p < 0.05; **p < 0.01; ***p < 0.001.

To test formal mediation, we used nonparametric bootstrapping to evaluate whether insomnia mediates the relationship between exposure to community weapon‐related violence and depression in adulthood (Table 2) (Hayes et al. 2017). The formal mediation analysis indicated a significant indirect effect of weapon‐related violence exposure on adult depression through insomnia symptoms (standardized indirect effect = 0.01, *p* = 0.01), while the direct effect remained significant (standardized direct effect = 0.09, *p* = 0.03). Overall, insomnia accounted for 7% of the total estimated effect of weapon‐related violence on depressive symptoms, thereby indicating a partial mediation effect.

## 8 Discussion

The results of this study support the hypothesis that insomnia is a pathway through which exposure to community weapon‐related violence in adolescence is associated with increased depressive symptoms in adulthood. Although insomnia accounted for a modest proportion of this association (7%), even small effects may have population‐level implications given the high prevalence of lifetime and repeated weapon‐related violence exposure. Nearly 1 in 4 adolescents (approximately 17.5 million adolescents) report lifetime exposure to weapon‐related violence (i.e., firearm or knife assault), and over 2 million have been directly victimized (Mitchell et al. 2015). Our findings corroborate prior research suggesting exposure to weapon‐related violence in adolescence is associated with depression (Clarke et al. 2020); specifically, as adolescent exposure to weapon‐related violence increases, their depression levels increase in adulthood. Notably, this association is held almost 13 years after exposure. Our findings underscore the association between community weapon‐related violence exposure during adolescence and depression in adulthood, with insomnia as a notable mediator. Furthermore, our study specifically focuses on violence involving weapons. The focus on weapon‐related violence is an important delineation from other forms of violence (e.g., physical violence) given the potentially heightened distress or trauma associated with weapon‐based victimization due to its lethal nature (Kagawa et al. 2020; Opara et al. 2020; Rajan et al. 2019). Additionally, our results are consistent with previous work showing that exposure to community violence can heighten the risk of insomnia among adolescents, subsequently contributing to poor mental health, including internalizing behaviors (Wright et al. 2017). In this study, we highlight a plausible pathway whereby insomnia is affected by violence exposure and, in turn, is associated with depressive symptoms.

## 9 Adolescent Weapon‐Related Violence Exposure and Insomnia

This study extends previous literature by employing longitudinal data to examine how weapon‐related violence exposure in adolescence can impact depression in adulthood through insomnia. Our findings indicated statistically significant mediation between weapon‐related violence exposure and depression. Moreover, adolescent weapon‐related violence was significantly associated with insomnia symptoms. This finding supports extant literature suggesting the negative impact exposure to weapon‐related violence in adolescence has on sleep among adolescents (Kliewer and Lepore 2015; Wright et al. 2017). Youth who reported witnessing a homicide were twice as likely to wake up after sleep onset compared to youth who did not (Spilsbury et al. 2014). However, after a 3‐month follow‐up, this relationship no longer remained statistically significant. Similarly, Wright et al. (2017) found that the relationship between exposure to community violence and sleep decreased as adolescents got older. While this could indicate adaptation and desensitization response from the effects of violence exposure over time, our study findings indicated a significant relationship after a 1‐year follow‐up after exposure to violence. However, the sleep effects could decline with follow‐up as adolescents get older, especially if adolescents experience chronic violence exposure.

Numerous studies have explored the impact of adolescent exposure to community violence on sleep, often employing cross‐sectional methodologies or shorter follow‐up durations (less than 3 months). However, our study assesses insomnia symptoms over 1 year following exposure to weapon‐related violence (extending up to 2 years post‐exposure). Investigating the effects of violence on sleep over a year post‐exposure is critical because adolescence is a sensitive period of development in which early experiences of violence can yield enduring and latent effects (Olofsson et al. 2012). This underscores the necessity for further research on the short‐ and long‐term effects of exposure to community violence on young, mid‐, and older adolescents.

Adolescents may develop sleep problems after a violent event due to experiencing psychological and physiological factors of stress (Heissel et al. 2018; Lynch et al. 2019). Exposure to weapon‐related violence is a stressor that can activate the biological stress response system (e.g., sympathetic nervous system and hypothalamic‐pituitary‐adrenal axis response) (Ford and Browning 2014; Suglia et al. 2015). A continuation of acute psychological stressors can increase the wear and tear on the body due to repeated activation of the response system, as well as cortisol levels (Koelsch et al. 2016; LaVeist et al. 2014). Elevated stress hormones can result in a state that adversely impacts sleep and insomnia, including wakefulness and sleep disruptions (Han et al. 2012). Although our study did not measure bio‐physiological indicators of stress, monitoring such levels in community violence‐exposed adolescents may help identify long‐term changes in physiological functioning as a result of weapon‐related violence exposure.

## 10 Insomnia Symptoms and Depression

Our study found a significant association between insomnia in adolescence and depression in adulthood. This finding aligns with the existing literature about the relationship between sleep problems and depression; sleep problems are strongly linked to depression in the future and are a primary risk factor for depression among those not currently depressed (O'Leary et al. 2017). Insomnia symptoms experienced for more than 2 weeks often predict an increased risk of developing depression within 1–3 years (Riemann 2003).

Both chronic and acute sleep loss and disruptions are linked to negative mood, heightened emotional reactivity (Payne and Kensinger 2011), and mental health outcomes among adolescents, including anxiety, major depression (Lovato and Gradisar 2014), ADHD (Hysing et al. 2016), impulse control disorders (Peach and Gaultney 2013), bipolar disorder, and suicide (Baldini et al. 2025). For example, Danielsson et al. (2013) found that sleep disturbances in high school students predicted depressive symptoms the following year. Inadequate sleep was also associated with multiple mental health physical symptoms including headaches and depressive symptoms, as well as emotional difficulties over time (Blake et al. 2018; Kelly and El‐Sheikh 2014; Vanek et al. 2020). One underlying factor that can explain the sleep‐depression pathway is the dysregulation of emotional reactivity (Baglioni et al. 2010), especially because disordered sleep has been linked to altered neurological functioning and decreased emotional expressiveness, and decreased impulse control (Peach and Gaultney 2013). This can lead to worse functioning over time, including the development of depression. O'Leary et al. (2017) found that maladapted emotional reactivity mediated the relationship between poor sleep and depression.

While there is prolific literature focusing on the relationship between sleep and depression, limited research has explored the nexus between sleep and depression over 12 years. Our study adds a distinctive dimension to this body of literature by examining the progression of insomnia during adolescence and its subsequent association with depression in adulthood over 12 years. This longitudinal examination of depression holds particular significance given that age‐related depression rates are highest in emerging and early adulthood (18–24 years old) (Lee et al. 2023). Further, our study offers a unique opportunity to examine insomnia symptoms a year after weapon‐related violence exposure but 12 years before depression, facilitating a better understanding of its potential role as a precursor to the development of depression. Nonetheless, future research should test various mechanisms that elucidate the pathways between poor sleep and depression, particularly from adolescence to adulthood.

An emerging body of literature suggests that the relationship between sleep problems and depression may be bidirectional—while insomnia can lead to depression, depression may lead to sleep problems (short sleep duration and sleep disturbances), which can, in turn, cause depression (Roberts and Duong 2013). However, a meta‐analysis found a stronger magnitude of effect from sleep problems to depression in adolescents instead of from depression to sleep problems (Lovato and Gradisar 2014). The risk of depression levels was higher when adolescents took a longer time falling asleep and spent more time awake in bed. Our study found that insomnia contributes to the development of depression, potentially due to exposure to community violence. This finding supports the directional relationship from insomnia to depression while also highlighting the possibility that a third, confounding variable is the cause of both maladaptive outcomes. In addition to the main mediation model where insomnia was tested as a mediator between weapon‐related violence and depression, we also tested a competing mediation model in which depression was the mediator between weapon‐related violence exposure and insomnia. This was done to explore the possibility of a cyclical relationship between depression and insomnia, as suggested by prior literature. The mediation analysis for this alternative model revealed a non‐significant indirect effect (*β* = 0.03, *p* = 0.16), indicating that depression does not significantly mediate the relationship between weapon‐related violence and insomnia. Compared to the competing model, these results strengthen the case that insomnia temporally mediates the relationship between adolescent weapon‐related violence exposure and adult depression, although it explains only a small portion of the total effect (partial mediation) and the large, significant direct effect indicates other pathways are also operative. Future research should more fully unpack the potentially bidirectional dynamics between sleep and depression; test additional mediators and moderators that could account for or shape the pathway; and incorporate resilience‐promoting factors, such as social support, adaptive coping, and community‐based resources, that can elucidate pathways between weapon‐related violence and depression and may buffer the impact of violence exposure on insomnia and subsequent depressive symptoms. Examining additional risk and protective mechanisms can inform strength‐based interventions to mitigate the mental health consequences of weapon‐related violence exposure.

## 11 Limitations

There are several limitations that should be considered in this study. First, the primary independent variable, exposure to weapon‐related violence, included both firearm and knife‐related injuries; only one item was firearm‐specific and asked whether the respondent was a gun violence victim. An alternative analysis was conducted using the one firearm‐specific item as the independent variable. However, while the point estimates were consistent in direction and magnitude with the original analysis, the mediation pathway was not significant.

The lack of delineation between weapons (firearms vs*.* knives) in questions about community violence exposure is a common limitation in the data that many researchers face, but nevertheless undermines the internal validity of the study. It thwarts the ability to test hypotheses about the differential experiences and effects of exposure to community violence. This is a critical research gap that warrants further research considering the fact that exposure to firearm violence can be an extreme form of violence that is more traumatizing than other forms of violence, including other weapon‐related victimization and non‐weapon‐related victimization (Kagawa et al. 2020; Rajan et al. 2019). Other limitations include the measurement of additional variables, such as the insomnia variable, which was not measured objectively. Subjective reports of sleep quality can be unreliable and susceptible to investigator effects. The measure of depression was also excessively brief and short‐term, only asking about emotional disturbances over the past week. More objective measures of depression would also be used longitudinally. Whilst both these limitations are a side effect of such a large, longitudinal study, they cannot be overlooked. Smaller, longitudinal studies using objective measures such as wrist‐worn actigraphy, wearable electroencephalography (EEG) devices, or polysomnography to capture nightly sleep duration, sleep efficiency (percent time in bed spent asleep), and wake‐after‐sleep‐onset (WASO) could confirm the results of this study with greater internal validity and reliability (Marino et al. 2013).

While the data from Waves 1 and 2 were from nearly 28 years ago, it remains germane today given the nationally representative sample and high rates of violence exposure among adolescents and their long‐term mental health effects. Adolescent rates of community violence exposure have remained high. Likewise, insomnia and other sleep complaints among adolescents have remained high in recent decades (de Zambotti et al. 2018; Uccella et al. 2023). Importantly, more recent longitudinal work (Nowakowski et al. 2016) has found similar pathways between violence, sleep disruption, and depression, all of which taken together suggest that our findings are relevant and inform today's prevention and sleep‐focused efforts. Nonetheless, the long‐time span poses challenges for generalizability because structural and economic contexts have changed in ways our models do not capture. Depression was assessed in 2008–2009 during the global financial crisis, which may have elevated symptom levels broadly; because that economic change was a common period exposure and our design is longitudinal, while it may have contributed to depression levels, it may be unlikely that this event fully explains the association between earlier weapon‐related violence and later depression. Nonetheless, these macroeconomic and other unmeasured structural factors remain potential confounders, and the findings should be interpreted with that caution within this limitation. Moreover, although the sample in this study is racially diverse, it may not be fully generalized to the broader adolescent population. The specific characteristics and experiences of the participants may differ from those of adolescents in other regions, neighborhood contexts, and demographic groups, potentially limiting the broader applicability of the findings. For instance, fatal school shootings are higher in rural and suburban schools, and handgun carriage is more prevalent in rural than urban counties (Livingston et al. 2019; Schleimer et al. 2023). Investigating these differences is critical, as elevated firearm homicide rates in rural settings may result in increased exposure to various forms of firearm violence (e.g., homicides, suicides), potentially leading to distinct insomnia and depression outcomes.

Lastly, we note the modest effect sizes observed in our mediation. While the associations are statistically significant, their magnitude is modest. This could be partly due to the data spanning several years (13 years), as is common in longitudinal studies where extended timeframes and multiple influencing factors may dilute the strength of individual associations. Nonetheless, the fact that a single event of weapon‐related violence exposure in adolescence remains associated with depression more than a decade later suggests that even small effects are meaningful, especially given the long follow‐up and the potential cumulative impact on the population level. Adolescents who experience repeated or cumulative weapon‐related violence (particularly in persistently high‐violence neighborhoods and during the peak victimization window in late adolescence when exposure tends to rise) are likely to experience greater sleep disruption and thus a larger indirect impact on depression than our estimates suggest (Lambert et al. 2005; Morgan and Oudekerk 2019). Nonetheless, even a small, mediated effect over such extended follow‐up can yield meaningful population‐level benefits, especially in light of today's higher rates of adolescent firearm violence exposure and insomnia symptoms (de Zambotti et al. 2018; Uccella et al. 2023). Moreover, because insomnia is modifiable through evidence‐based interventions, targeting sleep disruption could substantially reduce the deleterious impacts of adolescent violence exposure on long‐term mental health.

## 12 Future Research Directions and Prevention Implications

Despite these limitations, our findings address a research gap in understanding how community weapon‐related violence in adolescence can lead to depression in adulthood. The results suggest that insomnia, a year after exposure, is one pathway linking early violence to later depressive symptoms. This points to potential avenues for early mental health and violence prevention screening and intervention that address both the environmental factors contributing to community violence and the resulting mental health challenges, such as insomnia.

Adolescent insomnia is highly prevalent and often persists in adulthood (de Zambotti et al. 2018; Li et al. 2023). Taken together with our findings that even a single firearm violence incident can impact sleep and adulthood depression, screening for sleep difficulties is imperative for adolescents exposed to weapon‐related violence, especially for those residing in communities with high rates of violence.

As part of early identification, practitioners could incorporate brief, validated violence risk screeners such as the SaFETy score (Goldstick et al. 2017) and routinely take histories that include weapon‐related violence exposure to flag adolescents at elevated risk and tailor prevention efforts. Because sleep problems are modifiable through well‐validated interventions, targeting insomnia remains a promising strategy for reducing the long‐term mental health burdens of community violence. Our results highlight the opportunity for community organizations, local health services, and schools to prioritize effective interventions that specifically address sleep disorders, such as Cognitive Behavioral Therapy for Insomnia (CBT‐I) (Dewald‐Kaufmann et al. 2019). Early intervention during this sensitive developmental period may help mitigate depression in adulthood and other risks associated with sleep disruption.

Mental health and violence prevention programs can integrate sleep education initiatives that promote good sleep hygiene, identify insomnia symptoms, and provide strategies for managing community violence‐related stressors. Our findings highlight the potential for early, multifaceted interventions that tackle community violence exposures and sleep disturbances as interconnected challenges, combining individual and community‐level efforts to reduce the risk of depression in adulthood.

## 13 Conclusion

Our study found that insomnia partially mediates the relationship between weapon‐related violence exposure in adolescence and depression in adulthood, suggesting that such exposure is associated with later depressive symptoms, 13 years after exposure. This research contributes to the literature by clarifying the association between adolescent weapon‐related violence exposure, sleep problems, and depression in adulthood. Additionally, it highlights sleep interventions as a potential target for reducing depressive symptoms in adolescents exposed to violence. Further research is needed to better understand how cumulative exposure to weapon‐related violence affects long‐term mental health outcomes in adulthood.

## Funding

The authors have nothing to report.

## Ethics Statement

This study used data from the public‐use version of the National Longitudinal Study of Adolescent to Adult Health (Add Health), a publicly available, de‐identified dataset. The use of this dataset was exempt from additional ethics approval as it contains no identifying information about participants. The original Add Health study received ethical approval from the Institutional Review Board at the University of North Carolina at Chapel Hill, where the study was conducted.

## Conflicts of Interest

The authors declare no conflicts of interest.

## Supporting information

Supporting File

## Data Availability

The data that support the findings of this study are openly available in National Longitudinal Study of Adolescent to Adult Health at https://addhealth.cpc.unc.edu/. The data used in this study are from the public‐use version of the National Longitudinal Study of Adolescent to Adult Health (Add Health). These data are publicly available and can be accessed through the Add Health website (https://addhealth.cpc.unc.edu/).
