# Supplementary material for: The Long‐Term Impact of Adolescent Community Weapon‐Related Violence Exposure on Depression: Insomnia as a Mediating Pathway
Source: J Community Psychol. 2026 Jun 22;54(5):e70121. doi: 10.1002/jcop.70121 (PMC13285005; doi:10.1002/jcop.70121)
Supplement: Supplementary file 1 — Supporting File [file JCOP-54-0-s001.docx]

**Supplementary Tables and Figure**

## **Table 1S.**

## *Correlation Matrix for Primary Continuous Variables*

| Variable | 1 | 2 | 3 | 4 | 5 | 6 | 7 | 8 |
| --- | --- | --- | --- | --- | --- | --- | --- | --- |
| Depression (w4) | 1.00*** | 0.11*** | 0.03* | 0.01 | 0.11*** | -0.00 | -0.05** | -0.04* |
| Insomnia (w2) |  | 1.00*** | 0.08*** | 0.10*** | 0.05** | -0.10*** | -0.07*** | -0.00 |
| Smoking (w1) |  |  | 1.00*** | 0.61*** | 0.31*** | -0.24*** | -0.18*** | 0.00 |
| Smoking (w2) |  |  |  | 1.00*** | 0.37*** | -0.19*** | -0.27*** | 0.01 |
| Smoking (w4) |  |  |  |  | 1.00*** | -0.10*** | -0.12*** | 0.11*** |
| Alcohol (w1) |  |  |  |  |  | 1.00*** | 0.44*** | -0.11*** |
| Alcohol (w2) |  |  |  |  |  |  | 1.00*** | -0.06*** |
| Alcohol (w4) |  |  |  |  |  |  |  | 1.00*** |
| *Note.* Upper triangle only; Pearson correlation coefficients included for primary continuous variables. Insomnia is assessed at Wave 2; depression at Wave 4; smoking is days smoked in the past 30 days (Waves 1, 2, 4); alcohol is past-year drinking frequency (1 = every day/almost every day to 7 = never) at Waves 1, 2, and 4. Significance is indicated by the following: **p* < .05; ***p* < .01; ****p* < .001. | | | | | | | | |

**Table 2S.**

*Summary of Estimates for Variables Related to Depression*

| *Predictors* | *Wave* | *b* | *β* | *95% CI* | *p* |
| --- | --- | --- | --- | --- | --- |
| Violence exposure | 1 | 0.32 | 0.04 | [0.00, 0.07] | .03* |
| Insomnia | 1 | 0.15 | 0.07 | [0.05, 0.10] | < .001*** |
| Insomnia | 2 | 0.21 | 0.06 | [0.03, 0.09] | < .001*** |
| Depression | 1 | 0.26 | 0.26 | [0.23, 0.30] | < .001*** |
| Age | 1 | 0.03 | 0.01 | [-0.02, 0.05] | .42 |
| Sex | 1 | 0.25 | 0.04 | [0.00, 0.07] | .03* |
| Minority | 1 | 0.45 | 0.06 | [0.03, 0.10] | < .001*** |
| Household income | 1 | 0.00 | -0.03 | [-0.06, 0.00] | .08 |
| Father’s education | 1 | -0.13 | -0.04 | [-0.08, -0.02] | .003** |
| Alcohol | 1 | 0.04 | 0.03 | [-0.02, 0.05] | .31 |
| Alcohol | 2 | 0.00 | -0.02 | [-0.08, 0.08] | .16 |
| Alcohol | 4 | -0.01 | 0.00 | [-0.04, 0.02] | .61 |
| Smoking | 1 | 0.00 | -0.01 | [-0.05, 0.03] | .59 |
| Smoking | 2 | -0.01 | -0.04 | [-0.08, 0.00] | .07 |
| Smoking | 4 | 0.02 | 0.10 | [0.08, 0.15] | < .001*** |

*Note.* Estimates come from regression models predicting adult depressive symptoms. *b* = unstandardized coefficient; *β* = standardized coefficient; 95% CI refers to the confidence interval for the standardized estimate, which are rounded (actual lower bounds > 0). Violence exposure is coded 0 = none, 1 = any weapon-related exposure; insomnia and depression refer to symptoms at the indicated waves; sex and minority are dummy coded (reference = female; Non-Hispanic White, respectively); household income is in 1994 thousands; father’s education reflects highest attainment. Minority status includes respondents identifying as Black/African American or Hispanic. Significance indicators: **p* < .05; ***p* < .01; ****p* < .001.

**Figure 1S.**

*Mediation Model of Weapon-Related Violence Exposure on Adult Depression via Insomnia*

**
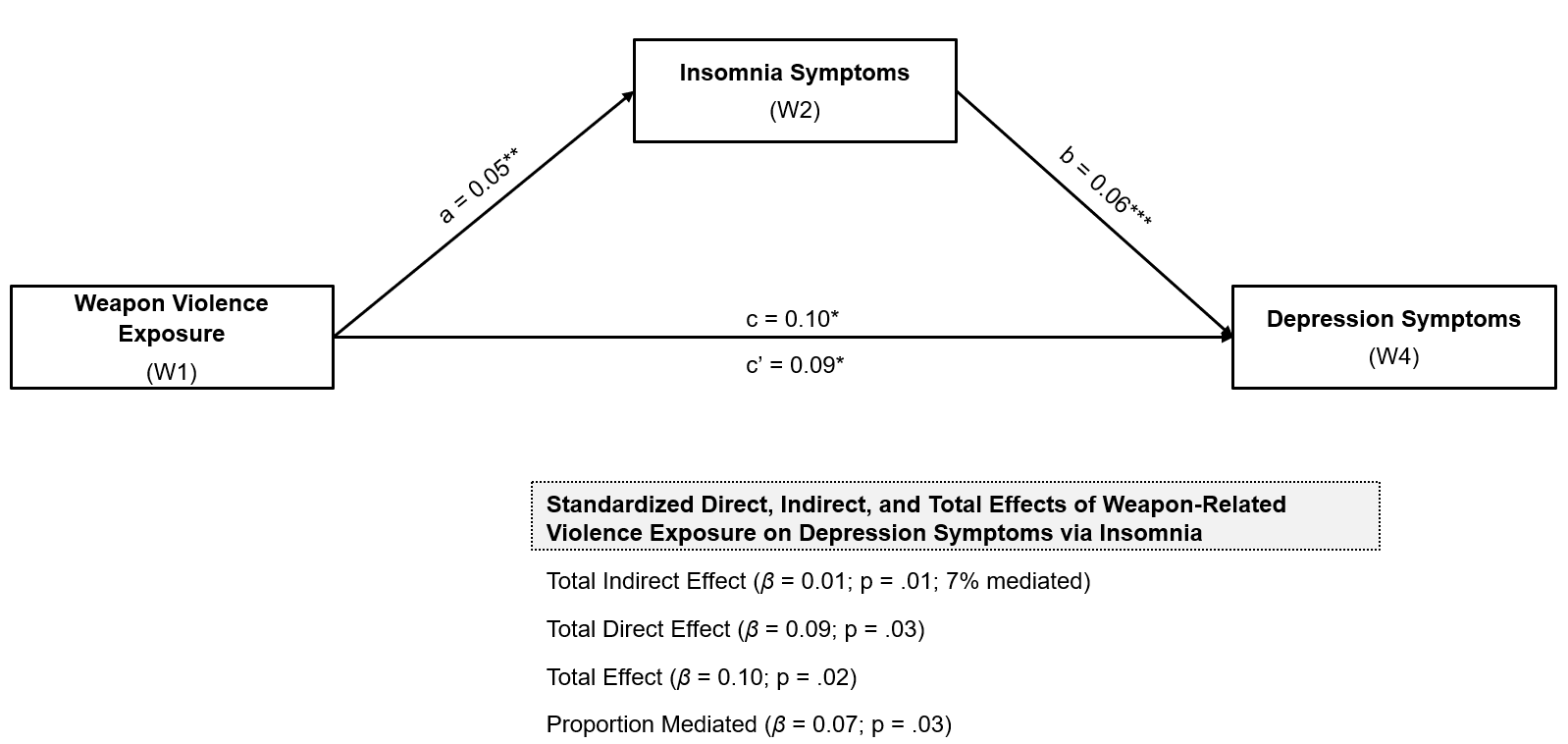
**

*Note.* Mediation model of weapon-related violence exposure in adolescence (Wave 1) on adult depression (Wave 4) through insomnia (Wave 2), adjusting for age, minority status (Non-Hispanic White vs. minority, including Black/African American and Hispanic), household income, father’s education, smoking, and alcohol. The a (0.05) and b (0.06) paths are standardized coefficients from the regression models; the total effect (c = 0.10), direct effect (c′ = 0.09), and indirect effect (β = 0.01; 7% mediated) are the total, average direct, and average causal mediation effects estimated via nonparametric bootstrap, such that total = direct + indirect. Insomnia and depression were z-scored prior to analysis. Confidence intervals are 95%; estimates are rounded (actual lower bounds > 0). Significance indicators: **p* < .05; ***p* < .01; ****p* < .001.
